# Quality of life of stroke survivors in Nigeria (Low‐income country). Can outcome be predicted?

**DOI:** 10.1111/jch.14258

**Published:** 2021-06-30

**Authors:** Gloria Adigwe

**Affiliations:** ^1^ School of Health and Bioscience University of East London London UK

**Story**

Stroke is the second leading cause of death and the third most common cause of long‐term disability worldwide. There are over 5.5 million deaths due to stroke and 44 million people who become disabled by it annually. The long‐term disability caused by stroke could lead to significant decline in the level of functioning and deterioration of quality of life. By 2025, the global population will equate to more than 800 million people of who two‐thirds are expected to live in developing countries. The literature identifies that there has been a decrease in stroke incidences worldwide in high‐income countries by 42% but in low‐income countries such as Africa there has been a 100% increase in stroke incidences. This has become an International concern as much is not known about the burden of stroke in Sub‐Saharan African. It has also become a huge public health issue worldwide and an important area for public health research. The story addresses the quality of life of stroke survivors in Sub‐Saharan Africa and Nigeria as little is known about this phenomenon.

**Significance**

Epidemiological studies of stroke in Africa particularly Nigeria have focused on mortality and risk factors profile but not on quality of life issues. Quality of life related to stroke and life satisfaction after stroke is important health care issues that have not received sufficient attention in Sub‐Saharan Africa. The term quality of life is defined as “an individual's perceptions of their position in life in the context of the culture and value systems in which they live and in relation to their goals, expectations, standards, and concerns.”

As the number of stroke survivors increases in Nigeria, it is essential to identify and modulate the factors that affect quality of life after stroke, particularly in the poorer communities, to promote maximal quality of life for stroke survivors in these poorer communities. It is imperative that every approach that can improve health care infrastructure and provide high‐quality rehabilitation programs should be considered. There is evidence that physical therapy that is conducted during rehabilitation of stroke patients reduces costs and improves quality of life “adjusted by years. The question for this story is—

“Quality of life of stroke survivors in Nigeria (Low‐income country) Can outcome be predicted?

The Health‐related quality of life in stroke patients – questionnaire is an instrument that has been developed for stroke survivors, it captures subjective realities of the survivor. It is argued that validated quality of life measures is necessary to improve the effectiveness rehabilitation programs for stroke survivors.

**Key Points**

The need to understand the impact of stroke on the quality of life of stroke survivors in Nigeria's poor communities is imperative. Surviving stroke can be a long‐term process that affects many aspects of a patient's life most especially if they live in a third world country. Ideally treatment for stroke should improve patient's quality of life by reducing the long‐term consequence of the event. Measurements of treatment efficacy that rely upon biological or clinical assessment do not always capture dimensions of heath that impact the quality of life of patients. Outcome measures and instruments are designed to measure health status form the perspectives of the patient, and usually include dimensions of physical health, social health, active daily living's, mental, and general health perceptions. Patient‐reported outcomes, such a health‐related quality of life, can capture subtle changes in health than are captured by traditional measures such as life expectancies. Evidence suspects that current outcome measures are insensitive to important determinants of life satisfaction for survivors after stroke.

Quality of life assessment is commonly incorporated in the overall evaluation of the impact of stroke, this could help both in knowing the areas in which a patient is most affected by the disease and in planning effective therapeutic and rehabilitative interventions. Furthermore, measurement of quality of life provides a meaningful way to evaluate the efficacy of stroke rehabilitation. Assessment of changes in quality of life during care may enable the evaluation of cost effectiveness to determine whether expenditures on health care are justified; hence, the use of the outcome instruments to help determine if outcome can be predicted.

## 1 MY PERSONAL “VIEWPOINT”

### 1.1 Quality of life after stroke in Nigeria (low‐income country) Can the outcome be predicted?

There has been increasing concern about the exponentially growing burden of non‐communicable diseases in Africa. Africa has, over the years, continued to transition from communicable diseases to non‐communicable diseases.^1^, ^2^, ^3^ The changes have been linked to improved medical facilities and services, improving basic amenities, and people's socioeconomic status in these regions.^4^ One of such non‐communicable diseases that has emerged as an outcome of the disease transition is hypertension which has resulted in stroke.^1^ The stroke cases among hypertensive patients have become a significant public health burden in Africa. Notably, in the Nigerian population, the commitment to stroke has increased the percentage of paralyzed patients in the country.^2^, ^3^

Globally, stroke is the second leading cause of death and the third most common cause of long‐term disability worldwide. There are over 5.5 million deaths due to stroke and 44 million people who become disabled annually. The developing countries are worse affected as the World Health Organisation (WHO) estimate suggested a sevenfold increase in disability‐adjusted life years attributable to stroke in low‐ and middle‐income regions compared to high‐income regions.^1^ This has become an international concern.^1^, ^4^, ^5^ Stroke, also known as a cerebrovascular accident (CVA), is a clinical syndrome rather than a homogeneous condition.

Stroke can be defined as “the sudden loss of blood supply to a region of the brain, leading to permanent tissue damage”.^6^ The most popular and widely used definition of stroke is that which was offered by the World Health Organisation (WHO) in 1978, which states that stroke is “a syndrome of rapidly developing clinical signs of focal or global disturbance of cerebral function, with symptoms lasting 24 h or longer, or leading to death, with no apparent cause other than of vascular origin”.

By 2025, the global population of this age group will be more than 800 million people, of whom it is expected that two‐thirds will live in developing countries. Sub‐Saharan Africa, particularly Nigeria, will be one area that falls into this category.

### 1.2 Public Health Burden of Stroke

Stroke is a disease of immense public health importance since it has economic and social consequences.^1^ It has become a substantial public health issue worldwide, particularly in low‐income countries, and a critical health research area.^1^, ^4^, ^7^ The burden of stroke does not lie in the high mortality figures, but the high morbidity, up to 50% of stroke survivors are chronically disabled.^8^, ^9^ Geographically, the burden of stroke varies widely, with the highest‐burden falling on Europe, North Asia, Central Africa, and the South Pacific.^8^ Until recently, stroke was a disease of the developed world. However, through the application of evidence‐based control measures and the implementation of various validated public health tools, the burden of stroke has been reduced drastically in many developed countries.^1^, ^10^ O’Donnell^11^ reports that between 1970 and 2008, there was a 42% decrease in stroke incidence in high‐income countries, whereas in low‐income regions such as Africa, particularly Nigeria, stroke incidence increased by 100%. Two‐thirds of stroke mortality cases occur in Africa,^10^, ^12^ where poverty, malnutrition, and communicable diseases such as HIV/AIDS are equally high. Data on Sub‐Saharan Africa show an annual stroke incidence rate of up to 316 cases per 100 000 people, a prevalence rate of 315 cases per 100 000 people, and a fatality rate of approximately 84%.^1^ The number of disability‐adjusted life years (DALYs) that is calculated to be due to stroke in Sub‐Saharan Africa is about seven times higher than that observed in high‐income countries. It is argued that in the next few decades, the burden due to stroke in Sub‐Saharan Africa is likely to increase substantially owing to the epidemiological transition in the region from infectious to non‐communicable diseases.^4^, ^8^

### 1.3 The Significance of this Study

Despite the pressure on public health caused by stroke in Nigeria, there are very few studies of the country's disease.^4^, ^13^, ^14^, ^15^, ^16^ Studies on the quality of life of stroke survivors are equally scarce, particularly in the southeast (SE) regions of the country,^8^, ^16^, ^17^ approximately 70% of the studies have been performed in the more affluent southwestern parts of the country.

Surviving a stroke can be a long‐term process that affects many aspects of a person's life. Stroke may lead to the most long‐term consequences of the event. Epidemiological studies of stroke in Nigeria have focused on mortality and risk factors profile but not on the quality of life issues.^18^, ^19^ Quality of life related to stroke is a significant health care issue that has not received sufficient attention in Nigeria..^8^, ^16^, ^17^ Stroke causes an adequate decrease in quality of life even among those with no residual effects..^12^, ^20^ Ideally, stroke treatment should improve patient's quality of life by reducing the long‐term consequences of the event. Measurements of treatment efficacy that rely upon biological or clinical assessment do not always capture health dimensions that impact the quality of life of patients. Health‐related quality of life instruments has been designed to measure health status from the patient's perspective. Patient‐reported outcomes such as health‐related quality of life can capture subtle changes in heath than traditional measures such as life expectancies.

Hamza et al^21^ state that a quality assessment is commonly incorporated into the overall evaluation of the impact on a survivor of stroke. Such an assessment helps to identify the survivor's areas of weakness. The World Health Organisation, Quality of life group (1996) and Owolabi^12^ report that the findings of an assessment may equally aid the stroke survivors as they plan effective therapeutic and rehabilitative interventions. Measurements of quality of life provide a meaningful way to evaluate the efficacy of stroke rehabilitation^22^, ^23^ and can also be used to evaluate the effectiveness of new health interventions.^8^ Further studies report that a comprehensive quality of life assessment facilitates a broad description of the disease and a range of problems that affect the patients and are essential from their perspectives.^13^, ^21^ Ultimately, assessment of the quality of life is paramount in health care practice and research, particularly in Nigeria, where evidence‐based medicine has become a vital priority of the Nigerian health care system. Therefore, studies designed to understand the nature and impact of stroke in the Nigerian population must contribute to managing the disease in the country.^13^, ^21^, ^22^

The work described in this thesis was intended to fill a gap in the literature concerning the quality of life of stroke survivors living in Nigeria's SE regions that will reflect on the global ethnic communities.

Thus, the aims of the study were:

- To determine the impact of stroke on the quality of life of survivors of the disease in Nigeria's SE communities.
- To explore perceptions, attitudes / cultural approaches, and knowledge of stroke and its determinants in the SE communities.

This mixed‐method cross‐sectional study will use convenience sampling. The study will be made up of two phases. Phase 1 will employ a quantitative approach and phase 2 a qualitative approach.

The impact of stroke will be measured using the health‐related quality of life in stroke patients’ tool. This is an outcome measure that employs a scale from 0 to 40. It was developed for use among stroke survivors; it has been validated and is deemed reliable for the assessment of quality of life after a stroke.^7^, ^14^

The perceptions, attitudes, and cultural approaches will be explored through the performance of semi‐structured interviews. This approach will aid in the detailed capture of the individual's perceptions concerning their quality of life. It is believed that this method would also uncover behaviors, trends in thought, and opinions of the participants to give further insight into the problem.^24^ A phenomenological approach captured by IPA (Interpretive phenomenological analysis) will be employed in this study in the analysis of qualitative. This is a recognized method that aims to offer insights into how a given person makes sense of a given phenomenon.^25^ It will allow patterns to emerge from the data to create themes.^26^ This method in the research will offer insights into the participants' cultural trends, perceptions, and experiences to provide a deeper understanding of the impact of a stroke. The analysis will involve a systematic protocol that will allow data to be deconstructed to facilitate the development of themes and involve four phases.

- Phase 1‐ Familiarization (Multiple reading and making notes)
- Phase 2 – Sensemaking (Transforming messages into Emergent themes)
- Phase 3 – Theory Building (Seeking relationships and clustering themes)
- Phase 4 – Data Refinement and Analysis (Writing up an IPA study)

As the number of stroke survivors increases in Nigeria, it is essential to identify and modulate the factors that affect quality of life, particularly in poor communities.^8^ To promote maximal quality of life for these patients, every approach that can improve health care infrastructure and provide high‐quality rehabilitation programs must be considered.

Ultimately, the study aims to identify the impact of stroke on the quality of life of stroke survivors by detecting the factors that could be examined to predict those changes. This will be achieved by measuring the differences and assessment of the relationships and/ or correlations found between the variables that are explored in different parts of the Health‐related quality of life in stroke patient instrument. This information will enable physiotherapists and allied health care professionals to improve their support for the needs of this group.

## CONFLICT OF INTEREST

None.
